# Supplementary material for: Scaling laws for rockfall impact fragmentation emerging from diverse lithologies
Source: Sci Rep. 2026 May 11;16:14735. doi: 10.1038/s41598-026-52503-w (PMC13161429; doi:10.1038/s41598-026-52503-w)
Supplement: Supplementary file 1 — Supplementary Information. [file 41598_2026_52503_MOESM1_ESM.pdf]

Scaling laws for rockfall impact fragmentation emerging from diverse lithologies

Álvaro Vergara, Sergio Palma and Raúl Fuentes

January 9, 2026

1 Particle Geometry Generation: Spherical Harmonics and Fractal Refinement

To accurately replicate the irregular morphology of the rock blocks used in this study, particle geometries were reconstructed using a Spherical Harmonic (SH) expansion, enhanced with fractal scaling to capture multi-scale surface roughness.

1.1 Spherical Harmonic Expansion

The surface of an irregular rock particle can be described by a radial function $r(\theta, \varphi)$, representing the distance from the particle's centroid to its surface at any given polar angle $\theta \in [0, \pi]$ and azimuthal angle $\varphi \in [0, 2\pi]$. This function is expanded into a series of spherical harmonic basis functions:

$$r(\theta, \varphi) = \sum_{n=0}^{\infty} \sum_{m=-n}^{n} c_n^m Y_n^m(\theta, \varphi) \tag{1}$$

where c_n^m are the complex SH coefficients that define the specific shape, and $Y_n^m(\theta, \varphi)$ are the Spherical Harmonic functions of degree n and order m, defined as:

$$Y_n^m(\theta, \varphi) = \sqrt{\frac{(2n+1)(n-m)!}{4\pi(n+m)!}} P_n^m(\cos\theta) e^{im\varphi} \tag{2}$$

where $P_n^m(\cos\theta)$ represents the associated Legendre polynomials:

$$P_n^m(x) = (1-x^2)^{|m|/2} \frac{\mathrm{d}^{|m|}}{\mathrm{d}x^{|m|}} \left[\frac{1}{2^n n!} \frac{\mathrm{d}^n}{\mathrm{d}x^n} (x^2-1)^n \right] \tag{3}$$

The low-degree coefficients ($n \leq 8$) primarily govern the macro-morphology (sphericity and elongation), while higher-degree coefficients represent the surface roughness.

1.2 Fractal Characterization of Roughness

To ensure the particles exhibit a "fractal nature" consistent with real lithologies (as proposed by Wei et al.), the SH coefficients are not assigned arbitrarily. The power spectrum of the coefficients, S(n), follows a power-law decay related to the Fractal Dimension (D):

$$S(n) = \sum_{m=-n}^{n} |c_n^m|^2 \propto n^{-\beta} \quad (4)$$

where β is the spectral exponent. This relationship allows the model to generate a population of particles where the roughness is statistically consistent across different scales, mimicking the multi-scale irregularities found in the Paleogene sandstones and Mesozoic limestones analysed in Catalonia.

The reconstructed geometries were discretised into polyhedral elements compatible with the Ansys Rocky DEM solver. The process ensures mass consistency (the volume of the SH-generated particle matches the target field block mass), and contact fidelity (the fractal roughness ensures realistic energy dissipation during the initial stages of impact, as the contact area evolves based on the local micro-morphology rather than simple spherical geometry).

The authors suggest the following references to support the SH theory:

- Zhou, B., Wang, J., and Zhao, B. (2015). Micromorphology characterization and reconstruction of sand particles using micro X-ray tomography and spherical harmonics. *Engineering Geology*, 184, 127-137.
- Wei, D., Wang, J., Nie, J. and Zhao, B. (2018). Generation of realistic sand particles with fractal nature using an improved spherical harmonic analysis. *Computers and Geotechnics*, 104, 1–12.
